# Negative Energy Balance Induced by Paradoxical Sleep Deprivation Causes Multicompartmental Changes in Adipose Tissue and Skeletal Muscle

**DOI:** 10.1155/2015/908159

**Published:** 2015-03-04

**Authors:** Marcos Mônico-Neto, Sara Quaglia de Campos Giampá, Kil Sun Lee, Camila Maria de Melo, Helton de Sá Souza, Murilo Dáttilo, Paulo Alexandre Minali, Pedro Henrique Santos Prado, Sergio Tufik, Marco Túlio de Mello, Hanna Karen Moreira Antunes

**Affiliations:** ^1^Departamento de Psicobiologia, Universidade Federal de São Paulo, Rua Napoleão de Barros 925, 04024-003 São Paulo, SP, Brazil; ^2^Centro de Estudos em Psicobiologia e Exercício, Rua Marselhesa 500, 04020-060 São Paulo, SP, Brazil; ^3^Departamento de Biociências, Universidade Federal de São Paulo, Rua Silva Jardim 136, 11015-020 Santos, SP, Brazil; ^4^Departamento de Bioquímica, Universidade Federal de São Paulo, Rua Pedro de Toledo 669, 04039-032 São Paulo, SP, Brazil

## Abstract

*Objective.* Describe multicompartmental changes in the fat and various muscle fiber types, as well as the hormonal profile and metabolic rate induced by SD in rats. *Methods.* Twenty adult male Wistar rats were equally distributed into two groups: experimental group (EG) and control group (CG). The EG was submitted to SD for 96 h. Blood levels of corticosterone (CORT), total testosterone (TESTO), insulin like growth factor-1 (IGF-1), and thyroid hormones (T3 and T4) were used to assess the catabolic environment. Muscle trophism was measured using a cross-sectional area of various muscles (glycolytic, mixed, and oxidative), and lipolysis was inferred by the weight of fat depots from various locations, such as subcutaneous, retroperitoneal, and epididymal. The metabolic rate was measured using oxygen consumption ($\dot{V}$O_2_) measurement. *Results.* SD increased CORT levels and decreased TESTO, IGF-1, and T4. All fat depots were reduced in weight after SD. Glycolytic and mixed muscles showed atrophy, whereas atrophy was not observed in oxidative muscle. *Conclusion.* Our data suggest that glycolytic muscle fibers are more sensitive to atrophy than oxidative fibers during SD and that fat depots are reduced regardless of their location.

## 1. Introduction

Studies have shown that rodents subjected to sleep deprivation (SD) protocols present substantial body weight loss [1–3]. Factors affecting body weight in these animals include altered hormonal profiles and feeding behavior and increased metabolic rate. In sleep-deprived animals, anabolic hormones such as testosterone (TESTO) [4], growth hormone (GH), insulin-like growth factor-1 (IGF-1) [5], and thyroid hormones T3 and T4 [6] are decreased along with increased activity of the hypothalamic-pituitary-adrenal axis (HPA axis) [1]. High levels of corticotrophin-releasing hormone (CRH) after 96 h of SD may contribute to considerable increase in metabolic rate and insufficient food ingestion, leading to weight loss [1, 7].

Koban and Swinson observed increased oxygen consumption ($\dot{V}$O_2_) in rats submitted to SD associated with weight loss and high levels of uncoupling protein-1 (UCP1), indicating a catabolic condition and high energy expenditure [8]. Such characteristics, observed in SD, generate a state of negative energy balance (NEB) [2]. NEB is defined as insufficient caloric consumption in relation to energy expenditure, creating a catabolic environment [7]. Sustained NEB results in muscle atrophy and lipolysis; therefore, white adipose tissue (WAT) and muscle trophism are good markers of the cellular metabolic state and energy balance [9–11].

A reduction of body fat and muscle has been documented in rats subjected to SD [2, 12]; however both tissues show distinct features according to the location and can respond differently to atrophic signal-induced SD. WAT depots differ from each other in the size of adipocytes, number and size of mitochondria, metabolic rate, and amount of enzyme (such as hormone-sensitive lipase (HSL) and adipose triglyceride lipase (ATGL)). Because of these peculiarities, some WAT depots may be more or less sensitive to glucocorticoids [13]. Only one study with SD observed fat reduction, but the authors observed it within a general context, without specifying the various fat depots. Various muscle fibers types may also respond to distinct forms of atrophic signals. Glycolytic fibers are more sensitive to high levels of glucocorticoids [14], as seen in cachectic syndromes such as chronic obstructive pulmonary disease (DPOC) [15], cancer [16], heart failure [17], and renal failure [18]; oxidative fibers are sensitive to microgravity [19], disuse, and denervation [20]. Predominantly, glycolytic muscle undergoes atrophy after sleep deprivation [12]. Therefore, an oxidative muscle could have a greater resistance to atrophic changes (induced by SD) than a glycolytic muscle.

Therefore, the aim of this study was to describe the multicompartmental changes in the fat and various muscle fiber types during SD-induced NEB and to correlate these changes with the hormonal profile and $\dot{V}$O_2_.

## 2. Materials and Methods

### 2.1. Animals and Housing Conditions

Adult male Wistar rats (3 months old, weighing 300–350 g) were provided by CEDEME (Centro de Desenvolvimento de Modelos Experimentais para Medicina e Biologia) of UNIFESP. The animals were housed in a temperature-controlled room at 22 ± 1°C, under a light-dark cycle of 12 hours with lights on at 7:00 am, and water and food were provided* ad libitum* throughout the experiment. All procedures used in the present study complied with the Guide for the Care and Use of Laboratory Animals, and the Ethics Committee of the Universidade Federal de São Paulo/Hospital São Paulo has approved the experimental protocol (#0764/10).

### 2.2. Experimental Protocol

Twenty animals were equally distributed into 2 groups: experimental group (EG) and control group (CG). Rats in the EG were sleep-deprived for 96 consecutive hours. CG rats were maintained in the same room as the EG, but without additional manipulation other than routine husbandry. Body weight was recorded daily between 8:00 and 9:00 am.

### 2.3. SD Procedure

SD was induced using a modified multiple platform method, as described by Suchecki and Tufik [21]. Rats were placed on circular platforms (6.5 cm diameter) located inside a water tank (123 × 44 × 44 cm). The upper part of the platform was kept 1 cm above the surface of the water. The animals were able to move around by walking from one platform to another. However, when they reached the paradoxical phase of sleep, the animals' faces would contact the water due to muscle atonia, and the animals would waken. All animals were adapted to the platform for 60 min during three consecutive days, and the SD started at 8:00 am of the next day. Chow pellets (Nuvilab) and water bottles were placed on a grid on top of the tank or home-cages. Previous studies show that the modified multiple platform method completely inhibits paradoxical sleep and causes a 37% decrease in slow-wave sleep [22].

### 2.4. Blood Sample Collection

After the SD or sleep periods, the rats were brought to an adjacent room in a random order and were decapitated. The blood samples were collected at the same time of day for all animals. They were collected during the morning, immediately after SD (between 8:00 and 10:00 am), to obtain plasma and serum, which were stored at −80°C until the assays were performed. Serum TESTO levels were measured by a chemiluminescent enzyme immunoassay (*Unicell DXI 800*,* Beckman Coulter*, USA). Plasma CORT concentrations were assayed using a double antibody radioimmunoassay specific for rats (MP Biomedicals, USA). Plasma IGF-1 was assayed using an ELISA kit (USCN Life Science, Germany). Serum T3 (triiodothyronine) and T4 (thyroxine) were measured by a chemiluminescent enzyme immunoassay (*Unicell DXI 800*,* Beckman Coulter*, USA). Glucose levels in plasma were measured by the glucose oxidase method (YSI 2300 STAT; Yellow Springs Instruments, Yellow Springs, OH, USA).

### 2.5. Tissue Processing and Histomorphometric Analyses

After blood collection, the* Tibialis Anterior* (TA),* Flexor Digitorum Longus* (FDL),* Gastrocnemius* (GASTRO), and* Soleus* (SOL) muscles from the right leg of each rat were excised, cleaned, and weighed. The distal half of each muscle was immediately frozen in hypercooled isopentane. All of the samples were stored at −80°C until the analyses were performed. Transverse sections (8 *μ*m thick) were cut from the mid-bellies of the muscles using a cryostat at −20°C, melted onto poly-*L*-lysine coated microscopy slides (Superfrost, Fisher Scientific, USA), and stained with hematoxylin-eosin (H&E). Digital images were taken from three to four H&E-stained sections derived from different animals (5 animals per group) using an Olympus BX50 brightfield microscope and a DP71 camera (Melville, NY) with a 40x objective. A blinded analysis of the cross-sectional area (CSA) of 200 fibers per muscle sample was performed using the Axio Vision 4.6 software (Carl Zeiss MicroImaging GmbH, Germany). To confirm the predominant features of the examined muscle fibers, the histochemistry for the ATPase enzyme was performed at pH 4.6.

After euthanasia, adipose tissue from the retroperitoneal, epididymal, and subcutaneous depots of the abdominal region was excised and weighed.

### 2.6. Metabolic Assessment

Other groups of animals were used for metabolic analysis by $\dot{V}$O_2_. The animals were distributed into two groups, EG and CG. They were previously adapted to a metabolic analysis apparatus for 1 hour, for 3 consecutive days. After the adaptation, the animals were kept for 7 days in the housing box and were transferred to the SD protocol for 96 h. At the end of the SD, the animals were individually placed in the metabolic evaluation apparatus for 24 h (sleep rebound). Metabolic assessment was performed using an indirect calorimetry system in open-circuit chambers (CLAMS; Oxymax open-circuit calorimeter, Columbus Instruments, Ohio, USA).

Sample air was passed through sensors for determination of the oxygen (O_2_) and carbon dioxide (CO_2_) content. The sensors were calibrated using a standard gas mix containing defined concentrations of O_2_, CO_2_, and N_2_, as described by Kennedy and colleagues [23]. The $\dot{V}$O_2_ was determined using the O_2_ and CO_2_ input and output concentrations. The data were collected every 15 s using the Oxymax Windows Software v.4.59. The values for $\dot{V}$O_2_ were analyzed using the software CLAMS Examination Tool (v2.2.1 CLaAX, Columbus Instruments, Ohio, USA).

### 2.7. Statistical Analysis

The Statistica 12 (StatSoft Inc., Tulsa, USA) software package was used for all statistical analysis, and the data are presented as the mean ± standard deviation (SD). Covariance analysis (ANCOVA) with Duncan's post hoc test was utilized to compare the muscle and adipose tissue weight and the muscle CSA. The tibia length (cm) was used as a covariate. The $\dot{V}$O_2_ analysis during sleep rebound was performed using variance analysis (two-way ANOVA), followed by Duncan's post hoc test, and for comparisons of hormonal data, the *t*-test was used. Statistical significance was accepted at *P* ≤ 0.05.

## 3. Results

**Figure 1:**
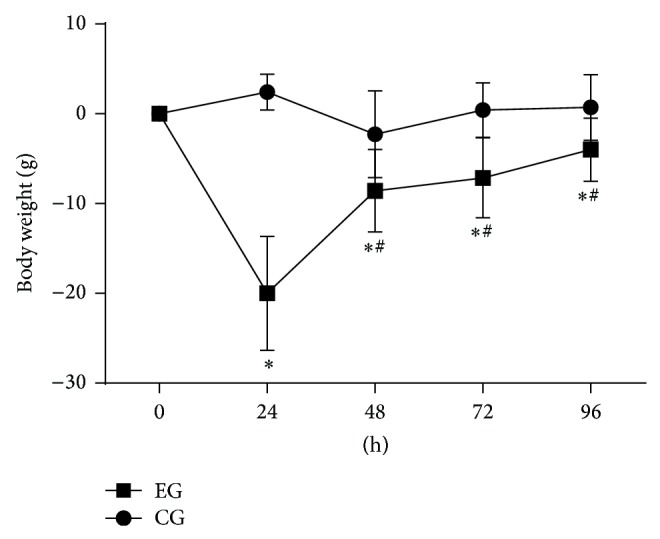
Weight variation during each 24 h of SD. Control group (CG), experimental group (EG). The data are shown as the mean ± standard deviation. (^*^) Difference between EG and CG and (^#^) difference from those 24 h, within the same group. *P* < 0.05.

SD reduced total testosterone (*P* < 0.01), plasma IGF-1 (*P* < 0.01), and T4 (*P* = 0.02) in EG compared with CG. In contrast, plasma corticosterone was increased in EG (*P* < 0.01). The T3 and glucose levels did not change (*P* = 0.3, Table 1). Animals from the EG showed reductions in mean body mass after SD (39 g, *P* < 0.01), whereas the CG did not show significant changes during the same time period. The EG showed high weight loss during the first 24 hours, with less weight loss after that period. All data points from the EG were different from the CG (*P* = 0.01, Figure 1).

**Figure 2:**
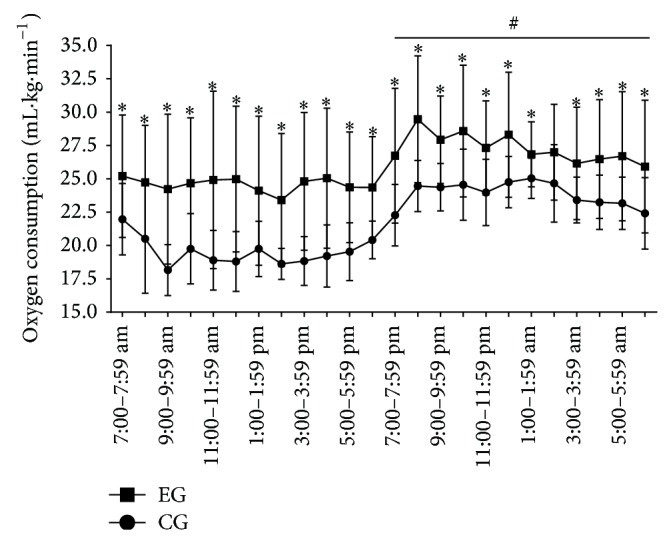
Oxygen consumption for 24 hours after the end of SD. (-■-) Experimental group (EG) and (-●-) control group (CG). The horizontal black line shows the dark period (7:00 pm–6:59 am). Data are shown as the mean ± standard deviation; statistical significance was accepted at *P* < 0.05; (^*^) difference between the CG at EG at each time point, (^#^) mean different between light and dark periods within each group.

**Figure 3:**
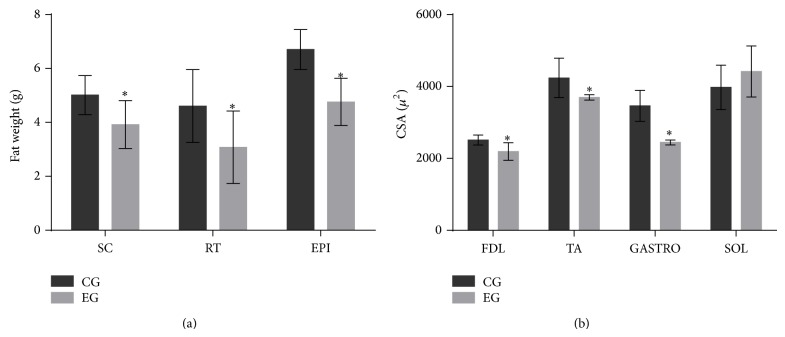
(a) Weight of subcutaneous (SC), retroperitoneal (RT), and epididymal (EPI) fat pads and (b) cross-sectional area (CSA) of the* Flexor Digitorum Longus* (FDL),* Tibialis Anterior* (TA),* Gastrocnemius* (GASTRO), and* Soleus* (SOL) muscles. The data are shown as mean ± standard deviation; statistical significance was accepted at *P* < 0.05. (^*^) Difference between the EG and CG.

The EG showed an increase in $\dot{V}$O_2_ during 24 h of rebound sleep (*P* < 0.01, Figure 2). The CSA in the FDL and TA (predominantly type II fibers) and GASTRO (predominantly mixed fibers) showed reduced muscle trophism (*P* < 0.01) in EG, whereas the SOL (predominantly type I fibers) did not change (*P* > 0.05) (Figure 3(b)). The adipose tissue from the three different compartments weighed less in the EG compared with the CG (*P* < 0.03, Figure 3(a)).

## 4. Discussion

In this study, we showed that SD decreased TESTO and IGF-1 and increased CORT levels, favoring a catabolic environment. Additionally, the EG showed an increased $\dot{V}$O_2_, reduced fat depots, and atrophy of glycolytic and mixed muscle. These changes, together with body weight loss, indicate that SD induces NEB.

The distinct atrophic responses of different muscles of rats subjected to SD can be explained by greater sensitivity of glycolytic muscles to glucocorticoids. High levels of CORT can activate the transcription factor forkhead box O (FoxO) and increase the activity of two major protein degradation pathways: the ubiquitin-proteasome and autophagy-lysosomal systems [24]. These pathways are known to be inhibited in slow-twitch fibers due to the high expression of peroxisome proliferator-activated receptor-g coactivator-1 (PGC1*α*), which inactivates FoxO, protecting slow-twitch fibers from atrophy [25].

WAT is divided into two major depots: subcutaneous and visceral fat. Subcutaneous fat forms the hypodermis, whereas visceral fat surrounds organs in the abdominal cavity and mediastinum [13]. Although each fat depot is functionally and structurally different and presents distinct responses to hormonal and sympathetic stimuli [13, 26, 27], we observed similar reductions in subcutaneous (abdominal region) and visceral (epididymal and retroperitoneal) fat during SD. The rise in CORT for a prolonged time during SD may explain the reduction of adipose tissue in rats, increasing the lipolytic rate in the EG by increasing ATGL expression and HSL phosphorylation [28]. Perhaps these adaptations occur because SD increases metabolic demand, as demonstrated by high $\dot{V}$O_2_, as a strategy to increase the availability of energy substrates, thus corroborating the findings of previous studies [2, 8]. The size of adipocyte varies in relation to the cell lipid content [13]. Although we have not measured the lipolytic rate using a direct method, we consider that the reduction in adipose tissue depots reflects lipolysis. However, because this is an indirect measure, it is inherently less precise and represents the major limitation in our study.

The adipose and muscle tissues are important sources of substrate during starvation and thus are good indicators of nutritional status. In the NEB, mobilization of fatty acids from adipose tissue is induced to replenish ATP sources via *β*-oxidation in muscle, liver, and heart [29]. In skeletal muscle, protein catabolism generates amino acids, which can be used as precursors for gluconeogenesis and ketogenesis [30]. This process is sensitive to lack of substrate and contributes to the maintenance of blood glucose levels in animals submitted to SD.

It is possible that the HPA axis guides the metabolic adaptation seen with SD. Tiba and colleagues administered a drug to inhibit CORT synthesis (metyrapone) to rats during SD for 96 h, and they saw that the drug inhibited body weight loss. Unfortunately, this study did not evaluate variations in skeletal muscle and specific fat depots [31]. The increase in HPA axis activity (more specifically CRH) could have an anorexic effect in the first 24 h of SD, as Martins and colleagues showed. This could be the reason for elevated body weight loss in this period of our study. The Martins et al. (2010) and Galvão et al. (2009) studies did not report the same anorexic behavior, although an increase in food intake was observed in the last 48 h of SD. These results could explain the lower rate of weight loss seen by us in the final 48 h. The high activity of the HPA axis stimulated the release of orexigenic neuropeptides, orexin, and neuropeptide Y and decreased levels of leptin, the major inhibitor of neuropeptide Y, thereby stimulating food intake [1, 3, 8].

In human studies, chronic disruption of the physiological light/dark cycle yields increased appetite and body weight, hormonal changes, fatigue, and psychological disorders [32]. In this study, the SD protocol maintained the habituated light/dark cycle (light between 07:00 am and 07:00 pm), exempting our results from bias due to perturbations in the circadian rhythm. The $\dot{V}$O_2_ could demonstrate oscillations in metabolic circadian rhythm, and this variable showed the same behavior between groups. Therefore, we believe that our results are induced by the stress of SD.

Thyroid hormones are known to increase $\dot{V}$O_2_ [33, 34]. However, in our study, no correlation was observed between T3/T4 and $\dot{V}$O_2_. A previous study showed that SD caused central hypothyroidism by posttranscriptionally downregulating the synthesis of thyrotropin-releasing hormone (TRH), which might be a compensatory response to an excessive increase in metabolic rate induced by the HPA axis. Reduction of TRH can induce the conversion of T4 to T3 to increase hormonal sensitivity through an increase in expression of the 5′-deiodinase type II (5′-DII), causing a reduction in T4 levels, as observed in our study [6].

In conclusion, our data suggest that SD induces a catabolic condition in fat depots, independent of their localization. In skeletal muscle, glycolytic fibers are sensitive to atrophy, and oxidative fibers appear to be more resistant to SD-induced atrophy signals.

## Figures and Tables

**Table 1:** Hormonal profile between control rats and sleep-deprived rats.

	CG	EG	*P*
TESTO (ng/dL)	259 ± 69	141 ± 29	≤0.001^*^
T3 (ng/dL)	58.5 ± 14	64.7 ± 14	0.17
T4 (ng/dL)	7.01 ± 2	5.02 ± 1.41	0.02^*^
IGF-1 (pg/mL)	274 ± 66	160 ± 36	≤0.001^*^
CORT (ng/mL)	74 ± 61	277 ± 132	≤0.001^*^
Blood glucose (mg/dL)	96 ± 25	103 ± 17	0.43

TESTO: total testosterone; T3: triiodothyronine; T4: thyroxine, IGF-1: insulin-like growth factor-1, CORT: plasma corticosterone; CG: control group; EG: experimental group. Data are shown as the mean ± standard deviation, ^*^ *P* < 0.05.
